# Electromagnetic Navigation System with a Marker Option for Computed Tomography-Guided Microwave Ablation of Undetectable or Inconspicuous Hepatic Tumors in Non-Enhanced Scans: A Feasibility Study

**DOI:** 10.3390/cancers18010025

**Published:** 2025-12-21

**Authors:** Myrto Papadopoulou, David Dimitrios Chlorogiannis, Ornella Moschovaki-Zeiger, Nikolaos-Achilleas Arkoudis, Athanasios Giannakis, Symeon Lechareas, Georgios Velonakis, Olympia Papakonstantinou, Dimitrios Filippiadis

**Affiliations:** 1 2nd Department of Radiology, University General Hospital “ATTIKON”, Medical School, National and Kapodistrian University of Athens, 12462 Athens, Greece; myrto96@gmail.com (M.P.); ddxlorogia@gmail.com (D.D.C.); m.z.ornella@gmail.com (O.M.-Z.); nick.arkoudis@gmail.com (N.-A.A.); a-giannakis@hotmail.com (A.G.); giorvelonakis@gmail.com (G.V.); sogofianol@gmail.com (O.P.); 2 Interventional Radiology Department, Faculty of Medicine, University of Thessaly, 41500 Larissa, Greece; slehareas@gmail.com

**Keywords:** ablation, computed tomography, liver

## Abstract

**Objectives**: Primary objective was to report the feasibility, safety and efficacy of percutaneous ablation of hepatic malignant tumors that are undetectable or inconspicuous in non-enhanced computed tomography (CT) scans using an electromagnetic navigation system with a marker option. Secondary objectives included the evaluation of technical parameters including the accuracy of needle placement, the number of control CT acquisitions, and procedural duration. **Methods**: This prospective study (performed from 1 March 2022 until 30 November 2024) included all patients with hepatic tumors (not visible or poorly defined on non-enhanced CT) who underwent percutaneous microwave ablation (MWA). Technical efficacy was assessed with contrast-enhanced CT immediately post-ablation, and oncologic outcomes (overall and progression-free survival) were evaluated with MRI at 1, 3, and 6 months. **Results**: Fifteen patients (12 males, 3 females; mean age of 66 years) with 16 tumors (median diameter of 15 mm) were treated in 16 sessions. Tumor types included hepatocellular carcinoma (*n* = 7), colorectal metastasis (*n* = 4), ocular melanoma (*n* = 1), neuroendocrine tumor (*n* = 1), intrahepatic cholangiocarcinoma (*n* = 1), and breast cancer metastasis (*n* = 1). Median procedure time was 53 min, scans number was nine, needle length was 12 cm, and median deviation was 1 mm. No complications were reported. Primary efficacy rate was 94% (15/16), rising to a secondary (assisted) technique efficacy of 100% after re-ablation (one session). During median follow-up of 23 months, local tumor progression-free survival was 100%; distant progression-free survival was 80%, and two patients (13.3%) died, one being cancer-related. **Conclusions**: Electromagnetic navigation with a marker option enables safe, accurate, and effective MWA of inconspicuous hepatic tumors, achieving excellent local control with favorable oncologic outcomes.

## Simple Summary

The aim of the present study is to report the feasibility and efficacy of percutaneous ablation of hepatic malignant tumors that are undetectable or inconspicuous in non-enhanced CT scans using an electromagnetic navigation system with a marker software. Overall, 15 patients with 16 tumors were treated in 16 sessions. Over a median follow-up duration of 23 months, no records of local tumor progression were identified among the treated lesions (100% local tumor progression free survival). The results of the current study support utilization of an electromagnetic navigation system with a marker software for tumors that are undetectable or inconspicuous in non-enhanced CT scans.

## 1. Introduction

According to multidisciplinary guidelines, utilization of imaging-guided ablation therapies like radiofrequency (RFA) or microwave (MWA) is a safe and liver-preserving treatment option for patients with primary or metastatic hepatic tumors [1,2,3,4,5]. Tumor visibility and anatomical location are crucial aspects affecting ablative therapy outcomes, enabling precise positioning of ablation probes for thorough treatment with adequate margins [6,7,8]. Challenges in tumor visibility or anatomic location of the tumor may require either in- or out-of-plane trajectories, which potentially could increase radiation exposure and pose risks of inaccurate needle placement and incomplete treatment [9,10]. A commercially available electromagnetic navigation system for percutaneous ablation procedures has been reported to provide in- and out-of-plane trajectories, while its utilization results in reproducible, increased accuracy along with decreased procedural duration and radiation dose [11,12,13,14,15,16,17,18,19,20,21]. However, some lesions are not visible on non-enhanced CT scans, primarily due to a lack of contrast with the liver tissue because of fatty liver disease or liver fibrosis. Real-time ultrasound and CT or MRI fusion imaging has been reported to result in precise targeting (95.6%), achieving successful ablation (90.2%) of inconspicuous target tumors [22]. Technical limitations hindering utilization of fusion imaging include the fact that ultrasound is an operator-dependent imaging modality subject to real-time variability, the technical issues with registration and co-localization, data synchronization issues and software limitations. Another reported approach is combining transcatheter CT arterial portography or transcatheter CT hepatic arteriography with percutaneous ablation for liver malignancies that were obscure on non-enhanced CT (87% primary technique effectiveness at 3 months) [23]. Furthermore, a commercially available electromagnetic navigation system combined with High-Frequency-Jet Ventilation has been used for percutaneous ablation of small hepatic tumors that were invisible on ultrasound and hard to reach in CT scans [13]. The aim of the present study is to report the feasibility and efficacy of percutaneous ablation of hepatic malignant tumors that are undetectable or inconspicuous in non-enhanced CT scans using an electromagnetic navigation system with a marker software.

## 2. Materials and Methods

### 2.1. Patient Selection

This observational study prospectively evaluated in the period from 1 March 2022 until 30 November 2024 all patients with primary or secondary malignant hepatic tumors that are undetectable or inconspicuous in non-enhanced CT who underwent percutaneous microwave ablation therapy under CT-guidance facilitated by a commercially available computer-assisted electromagnetic navigation system with a marker software (Imactis^®^ CT-Navigation™ GE Healthcare, Chicago, IL, USA). Inclusion criteria included patients of age ≥ 18 years old, with primary or secondary malignant hepatic tumors undetectable or inconspicuous in non-enhanced CT scans (but visible in arterial or portal venous phase of contrast-enhanced CT scan), coagulation parameters within normal limits, life expectancy of >3 months; all included tumors/patients should have been evaluable for the 6-month post-ablation follow-up. Exclusion criteria included non-compliance of patients, uncontrollable INR, systemic or local infection, and presence of a medical or psychiatric illness that would preclude informed consent or follow-up. No patients were excluded based on the criteria. All patients were evaluated and referred to for thermal ablation by a multidisciplinary tumor board including surgeon, medical and radiation oncologist and interventional radiologist.

### 2.2. Procedural Details

Every patient included in the current study was thoroughly briefed on the procedure, potential complications, and alternative surgical options. Written consent, acknowledging both the technique and participation in this study, was obtained from all participants. Decision for percutaneous ablation was obtained in a multidisciplinary tumor board meeting including hepatologists, medical, surgical and radiation oncologists as well as diagnostic and interventional radiologists. All patients underwent laboratory workup 24 h prior to the percutaneous ablation session. According to the directions provided by the Infection Division of Pathology Department of the hospital, prophylactic antibiotic (4 + 0.500 g Piperacillin + Tazobactam) was administered intravenously 60 min before MWA session, repeated twice within 24 h. All ablation sessions were performed by the same operator with more than 15 years of experience in percutaneous ablation and >5 years of experience in the electromagnetic navigation system. The MWA session was always performed in an inpatient setting, under local sterility and anesthesia (10 cc of 2% Lidocaine Hydrochloric for skin and subcutaneous tissues) and intravenous analgesia (1 g of paracetamol and 100 mg of tramadol diluted in 100 mL of normal saline during the procedure).

**Figure 1:**
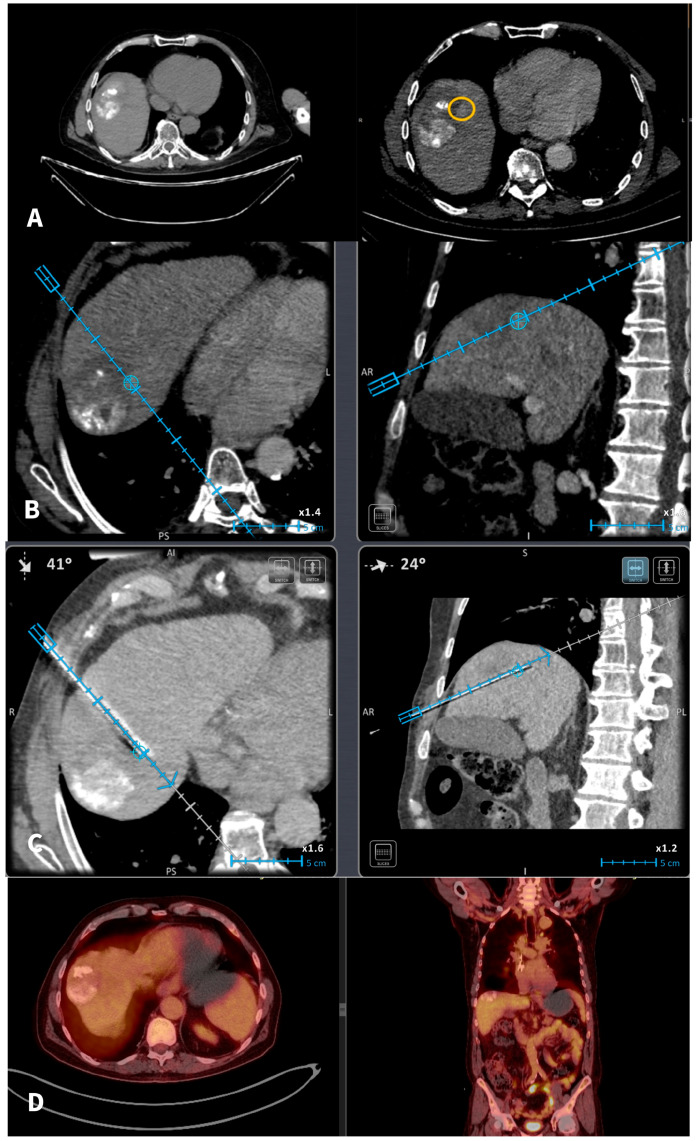
A 67-y-o male HCC patient who underwent in 2020 a combined therapy with MWA and transarterial chemoembolization. (**A**): In 2022 imaging follow-up depicts solitary hepatic lesion located in the hepatic segment VIII, 1 cm in diameter (recurrent HCC). The recurrent tumor was non-visible in the NCMCT scan. Axial CT scans pre and post-intravenous injection of Iodine contrast (portal venous phases) depicting the recurrent tumor (yellow circle) anteriorly and medially to the previously TACE + MWA zone of necrosis. (**B**): A commercially available electromagnetic navigation system with a marker software (blue circle) is used for tumor targeting and navigation of the microwave probe. (**C**): A commercially available electromagnetic navigation system with a marker software (blue circle) is used for navigation of the microwave probe. (**D**): Follow-Up PET-CT scan 6 months after treatment verifies total tumor necrosis.

The planning and placement of the microwave probe were carried out utilizing a commercially available electromagnetic navigation system designed for interventional radiology. As tumors were not visible in non-enhanced CT scans in all cases, iodinated nonionic contrast medium was intravenously injected; CT scans were obtained in arterial and portal venous phases in all patients. Subsequently, the series offering optimal tumor visualization on CT was transferred to the navigation system. Upon identification of the target lesion, a marker was placed on the screen of the navigation system of the exact size and at the exact location of the tumor target. The software provides the user with the option of moving the marker to the desired location and changing its size to meet the tumor characteristics. The additional value of marker option lies in the fact that, during the initial contrast-enhanced CT scan where the lesion is detectable, the marker can be used to delineate tumor’s location and size. In the subsequent scans where the tumor is not visible, the marker is there to delineate the target. Unfortunately, the marker is useful only for undetectable or inconspicuous hepatic tumors in non-enhanced CT scans, which are becoming visible in arterial or portal venous phase in CMCT. It was not able to be used until now in tumors that are not visible on CECT. In this study, all scans (both for setup and control) and needle movements were conducted during end-expiration apnea. A single microwave probe was used for ablating all tumors included in the current study. Once positioned accurately in the marker (i.e., tumor target), the ablation session was initiated and carried out based on coagulation charts provided by the manufacturer, considering factors such as tumor size, location, and the desired safety margin [Figure 1]. Goal of the ablation was a zone of necrosis including the tumor and minimum ablation margins of 5 mm for HCC and 10 mm for metastatic tumors. During probe removal from the liver in each session, track ablation was performed to minimize the risk of bleeding and tumor seeding. Post-ablation CT scans (pre and post-IV contrast medium injection in the arterial and portal venous phases) confirmed the ablation zone and assessed any immediate complications following the microwave ablation treatment. All patients were discharged from the hospital the following day.

### 2.3. Outcome Evaluation

The primary objective was to evaluate feasibility, safety and efficacy of performing percutaneous microwave ablation of tumors undetectable or inconspicuous in non-enhanced CT scans utilizing a commercially available electromagnetic navigation system with a marker option. Secondary objectives included the evaluation of technical parameters including the accuracy of needle placement, the number of control CT acquisitions, and procedural duration. Imaging and clinical follow-up consultation were performed at 1-, 3- and 6-month post-ablation with contrast-enhanced MRI. International guidelines for standard ablation terminology were used for definitions in the current study [24]. The reporting of complications was performed according to the modified Cardiovascular and Interventional Radiological Society of Europe (CIRSE) classification system [25].

## 3. Results

Baseline characteristics and demographics of patients and tumors included in the present study are presented in Table 1. Overall, 15 patients with 16 tumors were treated in 16 sessions. Of those, 71% (12/15) of patients were male with median age of 66 years (Interquantile Range [IQR]: 54–77 years). The median diameter of the lesions was 15 mm, and maximum tumor size ranged from 14 to 21 mm. Neoplasmatic substrate included hepatocellular carcinoma (7/15), colorectal cancer metastasis (4/15), ocular melanoma metastasis (1/15), neuroendocrine tumor metastasis (1/15), intrahepatic cholangiocarcinoma metastasis (1/15), and breast cancer metastasis (1/15). Tumor location was distributed among segments VIII (*n* = 8), VII (*n* = 5), and V (*n* = 3).

There was no need for hydrodissection or any other ancillary methods. The mean total duration of the procedure from entrance to exit of the patient was 53 min (IQR: 50–60 mins). A median of nine scans (IQR: 8–10) were performed including planning and control scans as well as a scan during ablation and immediate imaging follow-up with three scans (prior to and post-contrast medium injection in the arterial and portal venous phases). The median needle insertion length was 12 cm (IQR: 10–14 cm), while the median distance from the marker center was 1 mm (IQR: 0–1 mm). No complications or device-related adverse events were noted.

**Figure 2:**
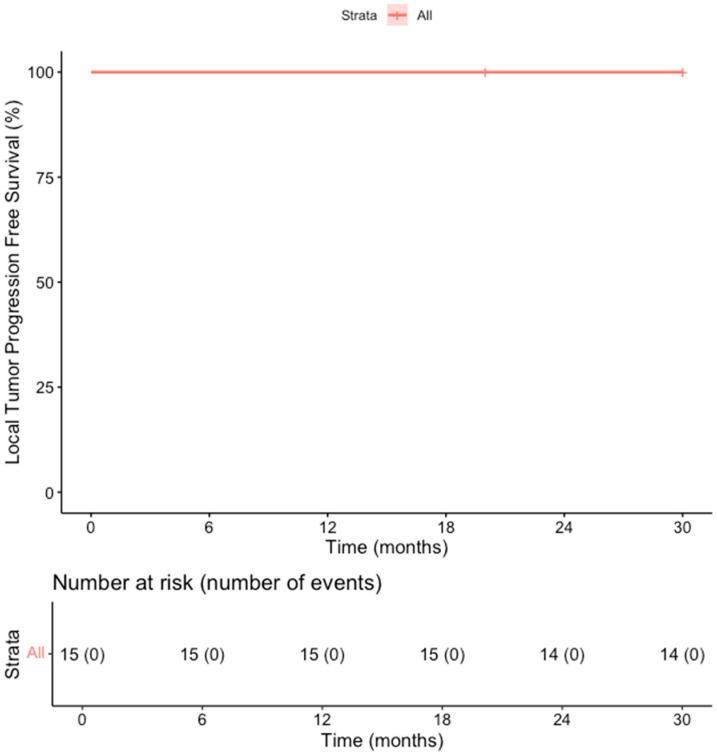
Kaplan–Meier Curve shows the Overall Survival in the overall cohort of patients.

**Figure 3:**
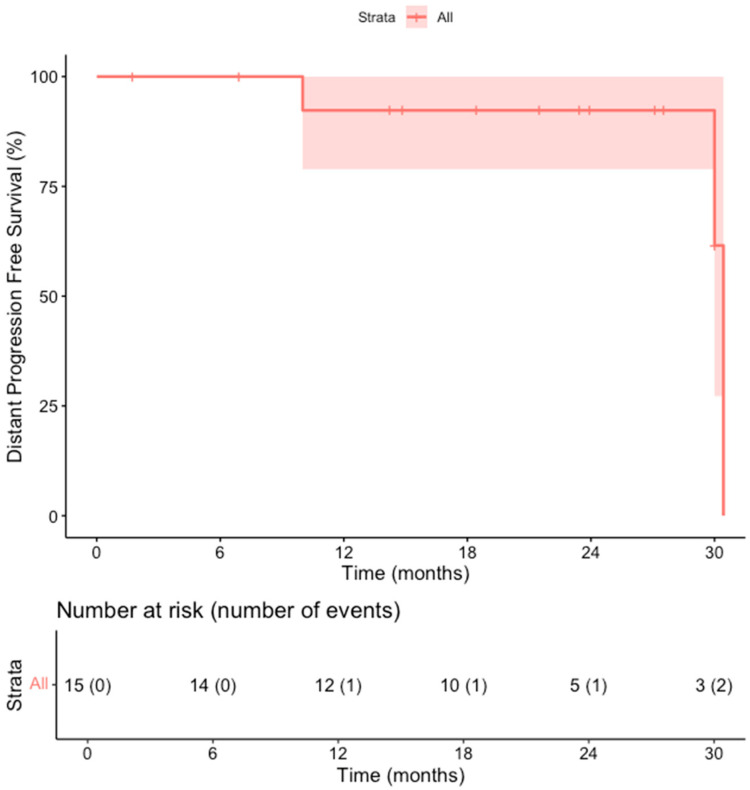
Kaplan–Meier Curve shows the distant progression-free survival in the overall cohort of patients.

**Figure 4:**
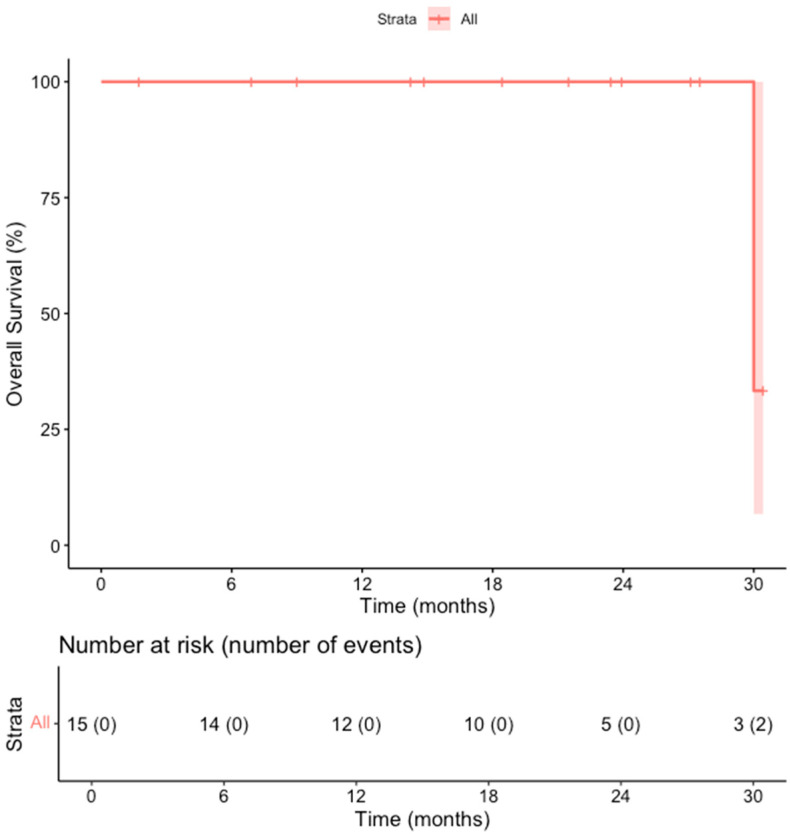
Kaplan–Meier Curve shows the local tumor progression-free survival in the overall cohort of patients.

The primary efficacy rate was 93.7% (15 out of 16 patients achieved complete ablation of the tumor, CI: 72–99%). There was evidence of a remaining viable tumor in the follow-up scan of one patient on the first follow-up MR scan 1.5 months post-ablation. This lesion was subsequently re-ablated for a secondary (assisted) technique efficacy of 100% (CI: 81–100%) after the second ablation session. Over a median follow-up duration of 23 months (IQR: 14–28 months), no records of local tumor progression were identified among the treated lesions (100% local tumor progression-free survival) [Figure 2]. However, disease progression was observed in 3 out of 15 patients (20%), reflecting the development of new lesions or advancement of disease outside the ablation zone (estimated distant progression-free survival at 24 months, 92% CI 79–100%). Specifically, one patient developed intrahepatic progression with the emergence of multiple new liver metastases 18 months after ablation, and two patients presented with metastatic spread to the lungs, bones, and liver 10 and 30 months after ablation, respectively [Figure 3]. Additionally, two patients (13.3%) died during the follow-up period, with one of these deaths attributed to cancer-related causes (the other patient developed hepatic decompensation characterized by liver failure and ascites secondary to underlying cirrhosis at 24 months post-ablation) [Figure 4].

## 4. Discussion

The results of the current study support utilization of an electromagnetic navigation system with a marker software for tumors that are undetectable or inconspicuous in non-enhanced CT scans. While this study demonstrates excellent clinical outcomes using electromagnetic navigation with marker-assisted targeting (15 out of 16 patients achieved complete ablation of the tumor), future integration with patient-specific multiphysics simulations similar to those exploring microwave ablation efficacy in early-stage hepatocellular carcinoma through finite-element modeling of electromagnetic energy deposition and thermal diffusion, could enable pre-procedural virtual ablation planning, margin optimization, and prediction of treatment success, particularly for inconspicuous or perivascular lesions [26].

The utilization of thermal ablation therapies for the treatment of primary and metastatic hepatic malignancies has been steadily increasing, driven by their minimally invasive nature, favorable safety profile, and efficacy in achieving local tumor control [1,2,3,4,5,27,28,29,30]. Achieving complete ablation remains a major challenge in the treatment of hepatic tumors. Factors such as tumor size and location, proximity to major vessels, the accuracy of probe placement, adequate thermal energy delivery, and proper assessment of the ablation zone all influence treatment success [6,7,8]. An additional and often overlooked difficulty is the limited visibility of certain tumors on non-enhanced CT scans, which can hinder precise targeting and post-procedural evaluation. This study adds to existing clinical evidence supporting the use of stereotactic navigation to guide percutaneous microwave ablation, highlighting its safety, feasibility, and effectiveness in managing malignant hepatic tumors—even under imaging limitations. Without a comparison to conventional CT-guided ablation or alternative targeting methods (e.g., CT-arterial portography, US/CT fusion), it is difficult to isolate the added value of the marker-assisted navigation system; however, the current study was a feasibility one focusing on preliminary results. Furthermore, it must be noted that the marker functionality relies on prior visualization of the tumor in contrast-enhanced phases, which excludes truly occult lesions (invisible even with contrast).

Volpi et al. reviewed 27 percutaneous ablations of small hepatic tumors that were invisible on ultrasound and hard to reach in CT scans, using electromagnetic navigation system combined with High-Frequency-Jet Ventilation reporting 96% needle placement accuracy in the first pass whilst complete ablation was observed in 100% of the cases at 6 months follow-up time; these results indicate that liver lesions that are not visible on ultrasound and require out-of-plane CT access can be effectively treated with percutaneous ablation using electromagnetic navigation and jet ventilation [13]. In the present study, computed tomography was also used as imaging modality of choice, and all treated tumors were undetectable or inconspicuous in non-enhanced CT scans; nevertheless, utilization of a commercially available electromagnetic navigation system with a marker option led to successful ablation in 15/16 target tumors (primary efficacy rate of 94%). In their study Volpi et al. reported a mean of a mean 23 ± 12 min for needle placement and a range of 1–7 control CT acquisitions (mean of 3.2). In the present study a mean of 53 min is reported, but that includes the total duration of the procedure from entrance to exit of the patient; the higher range of 8–10 control CT acquisitions (median of four scans) can be explained by the three scans prior (non-enhanced, arterial and portal venous phase) for tumor identifications and post-evaluation of the ablation results. Van Tilborg et al. studied 20 patients with unresectable liver malignancies that were obscure on non-enhanced CT to evaluate the feasibility of combining transcatheter CT arterial portography or transcatheter CT hepatic arteriography with percutaneous liver ablation; the reported results showed that single-session percutaneous tumor ablation guided by CT arterial portography or CT hepatic arteriography allows for repeated contrast-enhanced imaging and real-time contrast-enhanced CT fluoroscopy, enhancing the visibility of lesions [23]. The real added value of the marker option in the electromagnetic navigation system lies in the fact that software enhances visibility of tumors, which in non-enhanced CT scans are undetected or inconspicuous. The major advantage of combining transcatheter CT arterial portography or transcatheter CT hepatic arteriography with percutaneous liver ablation is that as a guiding modality it can be used in all liver tumors; on the other hand, the described marker option can be used only in cases where the target tumor is undetectable or inconspicuous in non-enhanced CT scan but becomes visible either in arterial or portal venous phase post-contrast enhancement.

This technology can easily be implemented for a wide variety of reasons including among others the fact that it is CT scanner-agnostic and requires a short learning curve. This fact outlines the streamlined workflow of the navigation system upon utilization in the CT suite. It is noteworthy that all ablation sessions and utilization of the electromagnetic navigation system with the marker option were performed under local anesthesia and IV analgesia; neither deep sedation nor general anesthesia was applied in the patients included.

The limitations of the present study include the small sample size and the absence of a comparison group undergoing alternative methods, such as CT arterial portography or transcatheter CT hepatic arteriography with percutaneous liver ablation. This small cohort limits statistical power and generalizability, particularly across diverse tumor types; however, these results are preliminary and hypothesis-generating, not definitive. Additionally, from an oncologic perspective, including patients with different types of malignancies reduces the reliability of the reported survival rates. Because there was no treatment failure across tumor types and locations, inter-tumor heterogeneity could not be assessed (ceiling effect), and the small per-type numbers leave the results susceptible to selection bias, confounding by indication, and misclassification bias. However, the main objective of this study was to assess the feasibility and technical success of using stereotactic navigation for CT-guided microwave ablation of malignant hepatic tumors that are invisible in non-contrast scans.

## 5. Conclusions

According to the results of the current study, utilization of an electromagnetic navigation system with a marker option during percutaneous microwave ablation of undetectable or inconspicuous hepatic tumors on non-enhanced CT scans is feasible, improves accuracy, and results in a safe and efficacious performance with satisfactory oncological outcomes. However, large randomized controlled trials are needed to evaluate the treatment’s effectiveness and to compare it with other available options.

## Figures and Tables

**Table 1:** Demographic and clinical data of the selected patients and lesions after MWA.

Demographics	Total Group
Patients (*n*)	15
Lesions (*n*)	16
Age (yrs)	66 (IQR 54–77)
Gender (M/F)	12/3
Tumor type	HCC [7/16] CLM [4/16] OMM [1/16] NETsM [1/16] ICCM [1/16] BCLM [1/16]
Tumor diameter (mm)	15 (IQR:14–21)
Tumor location (hepatic segment)	VIII [8/16) VII [5/16] V [3/16]

Note: IQR: interquantile range; HCC: hepatocellular carcinoma; CLM: colorectal liver metastasis; NETsM: neuroendocrine tumor metastasis; ICCM: intrahepatic cholangiocarcinoma metastasis; OMM: ocular melanoma metastasis; and BCLM: breast cancer liver metastasis.

## Data Availability

The data presented in this study is available upon request from the corresponding author due to privacy reasons.

## Abbreviations

The following abbreviations are used in this manuscript:

BCLM	Breast cancer liver metastasis
CIRSE	Cardiovascular and Interventional Radiological Society of Europe
CLM	Colorectal liver metastasis
CT	Computed tomography
HCC	Hepatocellular carcinoma
ICCM	Intrahepatic cholangiocarcinoma metastasis
IQR	Interquantile range
IV	Intravenous
MWA	Microwave ablation
NETsM	Neuroendocrine tumor metastasis
OMM	Ocular melanoma metastasis
RFA	Radiofrequency ablation
